# Health Information-Seeking Behavior in Older Adults with Vision Impairment Among Different Stages of Eye Care: A Cross-Sectional Comparative Study

**DOI:** 10.3390/geriatrics10040087

**Published:** 2025-07-01

**Authors:** Ya-Ping Wang, Ping Ouyang, Yan-Hua Zhao, Liu-Ming Lu, Hua-Ying Liu, Can Dai, Hong-Zhen Zhou

**Affiliations:** 1 School of Nursing, Southern Medical University, Guangzhou 510515, China; wangyaping@sz-eyes.com; 2 Department of Nursing, Shenzhen Eye Hospital, Shenzhen Eye Medical Center, Southern Medical University, Shenzhen 518040, China; 3 Department of Nursing, Nanfang Hospital of Southern Medical University, Guangzhou 510515, China

**Keywords:** vision impairment, older adults, health information, health behavior, eye care

## Abstract

**Background/Objectives**: Visual impairment (VI) represents a significant health challenge among older adults, particularly due to their limited understanding of health information. This study aimed to investigate and compare the health information-seeking behavior (HISB) of older adults with VI across different stages of eye care. **Methods**: A cross-sectional comparative study was conducted in 248 older adults with VI in various stages of eye care, including the pre-visit stage (*n* = 84), treatment stage (*n* = 83), and follow-up stage (*n* = 81) at the Shenzhen Eye Hospital from July to October 2024. Participants completed an HISB questionnaire encompassing four dimensions: attitude, needs, sources, and barriers. **Results**: The overall mean score of HISB varied significantly among the different eye care stages. The treatment stage had the highest scores (3.70 ± 0.32), particularly in attitudes toward health information and information needs. Poor self-reported overall health facilitated HISB in each stage. In the pre-visit stage, higher income was associated with increased HISB, while a lack of internet access reduced it. In the treatment stage, higher education was associated with higher HISB, whereas moderate-to-severe VI and certain income levels were associated with negative effects. In the follow-up stage, rural residence and spousal or child caregiving emerged as key facilitators of HISB. **Conclusions**: The treatment stage is a critical period for HISB engagement in older adults with VI. Healthcare providers should consider stage-specific factors of HISB to optimize health information delivery.

## 1. Introduction

Visual impairment (VI) is a prevalent sensory disability among older adults, with its prevalence increasing with age [1,2]. According to the Global Burden of Disease Study, in 2020, there were 33.6 million blind people aged 50 and over, 206 million with moderate-to-severe VI, and 143 million with mild VI [3]. As one of the largest and fastest-growing aging countries, China faces significant health burdens in managing older adults with VI [4,5]. The impact of VI encompasses multiple functional domains, including physical, psychological, cognitive, and social functioning, which are important contributors to the quality of life and healthy aging [6,7]. In conjunction with the provision of eye care services, comprehending how older adults with VI can access and utilize health information is essential as it affects their self-management capabilities, treatment adherence, and independence, thereby aiding in reducing the overall burden [8,9].

Health information-seeking behavior (HISB) typically involves individuals’ actions to obtain information regarding health conditions, treatments, and wellness practices, acting as a vital link between health needs and services [10]. Older adults with VI, impacted by aging and progressive eye conditions, often find themselves at a disadvantage when accessing health services and utilizing information, potentially facing the risk of blindness and complex decision-making [11]. Research indicates that effective health information-seeking enables patients to be better informed about their health conditions and treatment options, leading to enhanced health-promoting activities and improved clinical outcomes [12,13]. Consequently, healthcare providers must consider the information needs, preferences, and other characteristics of older adults with VI to ensure they benefit equally from healthcare services.

Previous studies have explored HISB in some contexts, such as coping with health issues, medical decision-making, and health-promoting behaviors [14,15,16,17]. Although individuals may exhibit stable health-seeking information propensities, HISB dynamically adapts to evolving personal and contextual factors [18]. Grounded in behavior change theories, information-seeking behaviors are shaped by interrelated processes: individual capabilities (e.g., self-efficacy), situational demands (e.g., decision urgency), and environmental resources (e.g., access to care) [19,20]. A comprehensive understanding of HISB thus requires an analysis of how these processes unfold across specific situations to fulfill information needs. Eye care stages can be complex, with patients potentially entering, leaving, and re-entering several times depending on their evolving eye conditions [21]. Typically, the entire eye care stage is described linearly with three key stages including pre-visit, treatment, and follow-up [21,22,23]. The three key stages align with key tenets of behavior change theories; information needs and behaviors are influenced by individual, situational, and environmental factors, which are likely to vary among these stages. However, there is still a notable gap in understanding how these behaviors manifest specifically among older adults with VI as they navigate through different stages of eye care [24].

This study aims to characterize and compare HISB in older adults with VI among different eye care stages, including pre-visit, treatment, and follow-up. Specifically, it focused on exploring the variations in attitudes, needs, sources, and barriers to health information among these stages and sought to identify the influencing factors contributing to these variations. The hypothesis was that HISB in older adults with VI will significantly vary among the three stages. This study is expected to provide insights into supporting and improving HISB among this population, which is crucial as healthcare approaches a more holistic, process-oriented, patient-centered care model.

## 2. Materials and Methods

### 2.1. Study Design and Participants

A cross-sectional study was conducted at the Shenzhen Eye Hospital, a municipal ophthalmic hospital in South China, from July 2024 to October 2024. People aged 60 years and older with bilateral or unilateral VI and a willingness to participate were recruited from a pre-admission center, a general inpatient ward, and an outpatient department. Participants could be in any phase of the three key eye care stages. According to the World Health Organization (WHO) criteria [25], VI was defined as presenting visual acuity (PVA, measured with spectacles or contact lenses if available) of <0.5 (decimal notation). Bilateral VI was assessed on PVA of the better eye, while unilateral VI was assessed on PVA of the worse eye [26]. Those with mental disorders, cognitive impairments, or severe hearing impairments who are unable to express themselves accurately or cooperate were excluded.

The sample size was calculated using multiple comparison corrections (Tukey–Kramer method) in PASS sample size software, version 2021 [27]. Based on the pre-survey results of the HISB score, the minimum detectable difference was set to 9 points and the standard deviation was set to 10. Assuming a 2-sided significance level of 0.05 and a power of 0.90 with an estimated 10% dropout rate, 73 participants were required in each group. This study included 248 participants, with at least 81 people in each group. The study adhered to the ethical standards outlined in the Declaration of Helsinki for biomedical research and was approved by the Ethics Committee of Shenzhen Eye Hospital, China (2024KYPJ088, 14 June 2024). All participants gave their informed consent in this study.

### 2.2. Instruments

Sociodemographic characteristics such as gender, age, educational level, place of living, primary caregiver, and monthly income were collected from all participants. Self-reported overall health and internet access were also queried. Additionally, information about VI regarding categories (unilateral or bilateral), duration, and severity levels in the better and the worse eyes were extracted from the medical records. Using decimal notation, VI severity was categorized as follows: (1) none as PVA ≥ 0.5; (2) mild as 0.3 ≤ PVA < 0.5; (3) moderate as 0.1 ≤ PVA < 0.3; (4) severe or blindness as PVA < 0.1 [25].

The HISB questionnaire was initially developed by Zamani et al. [28] and localized into Chinese by Sun et al. [29] to assess health information-seeking behaviors in chronic patients, including older adults. The instrument consists of 43 items in four dimensions: attitude to health information, information needs, information sources, and barriers to accessing health information. Each item was rated on a 5-point Likert scale of importance. Items 1 to 35 use positive scoring, while items 36 to 43 (barriers dimension) use reverse scoring. The higher the score, the more frequent the individual’s HISB and the greater its importance. The psychometric properties of the HISB questionnaire have been validated [29,30]. In this study, the Cronbach’s alpha for the 43 items was 0.891, calculated using SPSS 25.0. This indicated relatively high internal consistency despite multidimensional constructs and reverse-scored items. The participants were separated into high-level and low-level groups based on their median scores.

### 2.3. Data Collection

This study employed paper-based questionnaires with senior-friendly design elements, including 24-point large font typesetting, high-contrast ink printing, and a structured layout [31]. Accessibility aids (magnifying glasses, electronic text-to-speech devices) were provided on request, and all questionnaires were pretested in a pilot sample (*n* = 15) to optimize readability. For participants unable to complete the survey independently (due to severe vision loss or literacy limitations), trained investigators administered the questionnaire using a standardized proxy response protocol. While family members or friends were allowed to be present for emotional support, they were explicitly instructed not to influence responses, and investigators intervened promptly if suggestive comments were made.

### 2.4. Data Analysis

All statistical analyses were performed using IBM SPSS Statistics 25.0 software. Descriptive statistics were reported as means with standard deviations or numbers with percentages. The Chi-square test and Fisher’s exact probability method were used to analyze the differences in general characteristics of older adults with VI among the three-stage groups. One-way analysis of variance (ANOVA) and the Bonferroni method were used to compare the average scores of HISB among the three stage groups. Multivariable regression used binary logistic regression to identify potential factors that could significantly influence HISB in each stage. A two-tailed *p*-value lower than 0.05 was considered statistically significant.

## 3. Results

### 3.1. General Characteristics of Participants

Out of 262 approached participants, 248 questionnaires provided valid responses with a response rate of 94.66%. There were 84 participants in the pre-visit stage, 83 in the treatment stage, and 81 in the follow-up stage. Baseline characteristics among eye care stages are presented in Table 1. No significant inter-stage differences were observed in demographic or clinical variables (*p* > 0.05).

### 3.2. Comparisons of HISB Scores Among Different Eye Care Stages

The mean scores of HISB for participants among different eye care stages are shown in Table 2. The overall mean score of the HISB was (3.56 ± 0.32) in this study. The overall mean score of the HISB in the treatment stage was higher than that in the pre-visit stage and the follow-up stage, with a significant difference (*F* = 17.443, *p* < 0.001). In terms of the specific dimensions of HISB, the treatment stage showed higher mean scores for attitudes to health information and information needs compared to the pre-visit stage and the follow-up stage, with both comparisons being statistically significant (*p* < 0.001). For the dimension of sources of information, the pre-visit stage had a lower mean score than the treatment stage and the follow-up stage, with a significant difference (*p* < 0.001). The pre-visit stage showed higher mean scores for barriers to accessing health information compared to the treatment stage (*p* = 0.093) and the follow-up stage (*p* < 0.001).

Specifically, regarding attitudes to health information, participants believed that HISB positively increased hopefulness, especially for the treatment stage (4.31 ± 0.68). In the follow-up stage, the emphasis was on finding the best treatment (3.89 ± 0.92) and decreasing anxiety (3.89 ± 0.89). Finding reliable physicians and finding appropriate treatment methods were the top two significant information needs in each stage. Physicians, nurses, and family were the top three significant sources of information in each stage. The lack of staff’s accountability was the top-ranked barrier in the pre-visit stage and the follow-up stage; being not healthy enough to seek information, uncertainty about received information, and lack of familiarity with medical terminology were major barriers in the treatment stage.

### 3.3. The Influencing Factors Associated with HISB

After conducting a univariate analysis of the included variables, there were three variables (monthly income, self-reported overall health, and Internet access) in the pre-visit stage, six variables (education level, place of living, monthly income, categories of VI, VI in the better eye, and self-reported overall health) in the treatment stage, and three variables (place of living, primary caregiver, and self-reported overall health) in the follow-up stage. These were subsequently included in the binary logistic regression analysis for multivariable modeling (Table 3). As a result, we found that a monthly income of CNY 2000–6000 (OR, 3.73; 95% CI, 1.08–12.91; *p* = 0.037) and poor self-reported overall health (OR, 5.10; 95% CI, 1.46–17.90; *p* = 0.011) were positively associated with the high level of HISB in the pre-visit stage while not having Internet access (OR, 0.17; 95% CI, 0.04–0.68; *p* = 0.013) was negatively associated with the high level of HISB. For the treatment stage, a monthly income of CNY 2000–6000 (OR, 0.13; 95% CI, 0.03–0.57; *p* = 0.007), bilateral VI (OR, 0.16; 95% CI, 0.03–0.81; *p* = 0.026), mild VI (OR, 0.11; 95% CI, 0.02–0.62; *p* = 0.013), and moderate to blindness (OR, 0.16; 95% CI, 0.03–0.81; *p* = 0.026) in the better eye were negatively associated with the high level of HISB while education level of high school or above (OR, 6.45; 95% CI, 1.34–30.98; *p* = 0.020) and poor self-reported overall health (OR, 7.68; 95% CI, 1.45–40.64; *p* = 0.017) were positively associated with the high level of HISB. For the follow-up stage, living in rural areas (OR, 8.18; 95% CI, 1.15–57.91; *p* = 0.035), spousal caregiver (OR, 6.36; 95% CI, 1.14–35.40; *p* = 0.035) and child caregiver (OR, 10.64; 95% CI, 2.14–53.02; *p* = 0.004), and general (OR, 13.74; 95% CI, 1.89–99.79; *p* = 0.010) and poor (OR, 67.81; 95% CI, 9.73–472.69; *p* < 0.001) self-reported overall health were facilitating factors associated with the HISB.

## 4. Discussion

This cross-sectional study examined the HISB of older adults with VI, providing a stage-specific analysis among the pre-visit, treatment, and follow-up eye care stages. The findings revealed significant differences and unique influencing factors at each stage, highlighting the varied nature of HISB and the potential impact of stage-specific factors on these behaviors. This study extends previous research on HISB to allow healthcare professionals to understand how health information needs and behaviors differ for older adults with VI at distinct points in their eye care journey and to focus on the particular stage of contribution to eye care.

The results indicated that older adults with VI in the treatment stage demonstrated greater HISB engagement than those in the pre-visit and follow-up stages, especially reflected in their positive attitudes and high needs for health information. It suggested that older adults with VI in the treatment stage were more actively engaged in HISB when faced with critical decision-making and management processes, aligning with previous research [32]. Healthcare providers should leverage this treatment-stage peak in information receptivity. For example, they could implement shared decision-making tools, such as visual aids with large print or audio-recorded treatment options, to facilitate informed choices [33]. In addition, the overall mean score of the HISB in each stage was moderately high—slightly higher than that in older adults with systemic chronic disease [34,35]. This implies that the specific challenges associated with VI may lead to a greater reliance on health information, highlighting the distinctive nature of seeking health information in this population [36].

In the pre-visit stage, older adults with VI had fewer information sources but fewer barriers to accessing information. This highlights the need for enhanced patient education and awareness programs before the initial visit. Healthcare providers should focus on pre-visit information services, such as providing basic information about eye conditions and available services, and consider the pre-visit stage’s reliance on the Internet for information gathering. Consistent with previous findings [29,30], physicians and nurses ranked as the top two significant sources of information in this study, reflecting their pivotal roles in providing medical expertise and guidance [37]. In contrast to other populations, family members were identified as the third significant source of information for older adults with VI, surpassing healthcare organizations or traditional mass media [28,29,30]. This finding might be attributed to the unique blend of emotional support, trust, and personalized assistance that family members provide, which is particularly valuable for a vulnerable population like older adults with VI [38]. This insight suggests that healthcare providers should acknowledge and incorporate the role of family members in the health information dissemination process, ensuring that they are equipped with accurate and relevant information to support their loved ones.

The identified barriers to health information access among older adults with VI at various stages of eye care further highlight the need for a stage-specific, patient-centered approach to health education and support [39]. In the pre-visit and follow-up stages, the lack of staff accountability emerged as the top barrier. This finding emphasized the importance of strengthening accountability within eye care teams to ensure that patients were not only well-informed but also felt valued and supported [40]. Moreover, not being healthy enough, uncertainty about receiving information, and unfamiliarity with medical terminology were the main barriers to accessing health information in the treatment stage. It indicated that healthcare providers should carefully consider the timing and format of health information provision, ensuring accessible and timely communication strategies tailored to individuals’ varying health statuses in treatment [41]. A common barrier in the three stages for older adults with VI was a lack of knowledge about reliable information sources, especially in the follow-up stage. This barrier was consistent with the broader challenges older adults face in the digital information age, which highlighted a continuous need for education and guidance on health literacy [42,43].

Factor analysis associated with HISB among older adults with VI provided a critical understanding of the complex interplay between various influencing factors at different stages of eye care. Regarding general demographic characteristics, the most central predictors for HISB are age, education, gender, and financial income, as shown in the previous literature [44]. In this study, however, no significant differences were found in terms of age and gender. This might be attributed to the specific characteristics of this study population, consistent with the research findings in older adults with chronic diseases [34,35]. The observed shift in the association between a monthly income of CNY 2000–6000 and a high level of HISB from a positive correlation in the pre-visit stage to a negative correlation in the treatment stage revealed their focus and priorities change. It could imply that older adults within this specific income range might have different priorities or access to resources during treatment. In the treatment stage, the education level of high school or above was positively associated with a high level of HISB, suggesting that the better-educated may have greater information literacy and awareness of the importance of seeking health information and actively engaging in discussions with healthcare providers [35,45]. In the follow-up stage, those living in rural areas and with their spouse or children as the primary caregivers were positively related to a high level of HISB. Rural areas often lack convenient access to professional eye care services, and the follow-up stage requires continuous monitoring and management of the condition [9]. This means that rural-dwelling older adults might rely more on self-initiated information-seeking [46]. Spousal or child caregivers play an active role in facilitating information acquisition. This finding emphasizes the significance of considering both environmental and social support factors in understanding and promoting HISB among older adults with VI, especially those in the crucial follow-up phase of their eye care journey.

Poor self-reported overall health as a facilitating factor associated with the HISB in each stage indicates that self-reported overall health status should be considered when providing healthcare services and support. Those who perceive their health as poor are more likely to be actively engaged in information-seeking activities in an attempt to better understand, manage, and potentially improve their situation [23]. In the pre-visit stage, the negative correlation between not having Internet access and a high level of HISB was observed, while no significant correlation was found in the treatment and follow-up stages. This finding is consistent with other studies on online HISB [20,42], which also identified the pre-visit period as a crucial time when patients rely heavily on the Internet for gathering health information. In the treatment stage, it was found that older adults with bilateral VI and those with VI in the better eye had lower HISB. This could suggest that the severity of VI during this stage might limit their ability to access and process health information effectively [26,43]. It also implies that healthcare providers need to be more attuned to the specific needs of this group and consider alternative methods of information delivery, such as providing audio-based information or more detailed verbal explanations [31,47]. Additionally, it may highlight the importance of involving caregivers or family members in the information dissemination process to ensure that these patients are not left behind in understanding and participating in their treatment due to their vision condition.

There were certain limitations in this study. Firstly, as a cross-sectional study, it cannot establish causality between the identified factors and HISB, which prevents the determination of temporal sequence and causality in the observed associations. Secondly, the reliance on self-reported data introduces potential biases. Thirdly, it is a single-center study in Shenzhen, China, limiting the generalizability of findings to diverse cultural or healthcare settings. Future research should consider a longitudinal design or multi-center studies to better understand the dynamics of HISB and its determinants over time, deepen understanding of the mechanisms underlying these associations, and develop more effective interventions to optimize health information-seeking behavior.

## 5. Conclusions

In conclusion, this study comprehensively characterized and compared the HISB of older adults with VI among the pre-visit, treatment, and follow-up stages of eye care. The stage-specific differences in HISB and associated factors were identified, providing valuable insights for healthcare providers. The treatment stage, in particular, is marked by increased engagement in HISB, signaling a critical period for information dissemination. The interplay of demographic characteristics, health status, and access to resources with HISB is stage-dependent, underscoring the complexity of patient information requirements. Therefore, healthcare providers should consider these stage-specific variations and associated factors when formulating care plans and health information dissemination strategies.

## Figures and Tables

**Table 1:** General characteristics of the participants among different stages of eye care.

Characteristics		Pre-Visit Stage (*n* = 84) *n* (%)	Treatment Stage (*n* = 83) *n* (%)	Follow-Up Stage (*n* = 81) *n* (%)	Chi-Square	*p*-Value
Gender	Male	34 (40.5)	35 (42.2)	30 (37.0)	0.467	0.792
	Female	50 (59.5)	48 (57.8)	51 (63.0)		
Age (years)	60–69	36 (42.8)	33 (39.7)	31 (38.3)	1.840	0.765
	70–79	35 (41.7)	37 (44.6)	32 (39.5)		
	80–90	13 (15.5)	13 (15.7)	18 (22.2)		
Education level	Primary school or below	35 (41.7)	26 (31.3)	25 (30.9)	4.995	0.288
	Middle school	22 (26.2)	33 (39.8)	26 (32.1)		
	High school or above	27 (32.1)	24 (28.9)	30 (37.0)		
Place of living	Urban	46 (54.8)	55 (66.3)	39 (48.2)	6.369	0.173
	Town	23 (27.4)	15 (18.1)	27 (33.3)		
	Rural	15 (17.8)	13 (15.6)	15 (18.5)		
Primary caregiver	Spousal caregiver	37 (44.0)	39 (47.0)	22 (27.2)	8.354	0.079
	Child caregiver	32 (38.1)	32 (38.5)	39 (48.1)		
	others	15 (17.9)	12 (14.5)	20 (24.7)		
Monthly income (CNY, ¥)	≤2000	26 (31.0)	24 (28.9)	19 (23.5)	8.521	0.074
	2001~6000	30 (35.7)	43 (51.8)	32 (39.5)		
	>6000	28 (33.3)	16 (19.3)	30 (37.0)		
Categories of VI	Unilateral	20 (23.8)	22 (26.5)	26 (32.1)	1.476	0.478
	Bilateral	64 (76.2)	61 (73.5)	55 (67.9)		
VI in the better eye	None	20 (23.8)	22 (26.5)	26 (32.1)	2.755	0.600
	Mild	32 (38.1)	26 (31.3)	29 (35.8)		
	Moderate to blindness	32 (38.1)	35 (42.2)	26 (32.1)		
VI in the worse eye	Mild	23 (27.4)	11 (13.3)	17 (21.0)	8.722	0.068
	Moderate	31 (36.9)	25 (30.1)	25 (30.9)		
	Severe to blindness	30 (35.7)	47 (56.6)	39 (48.1)		
Duration of VI	≤1 year	40 (47.6)	47 (56.6)	36 (44.4)	2.632	0.268
	>1 year	44 (52.4)	36 (43.4)	45 (55.6)		
Self-reported overall health	Good	26 (31.0)	18 (21.7)	27 (33.4)	9.355	0.053
	General	23 (27.3)	30 (36.1)	13 (16.0)		
	Poor	35 (41.7)	35 (42.2)	41 (50.6)		
Internet access	Yes	18 (21.4)	14 (16.9)	12 (14.8)	1.301	0.522
	No	66 (78.6)	69 (83.1)	69 (85.2)		

Abbreviations: VI, vision impairment.

**Table 2:** Health information-seeking behavior scores among different stages of eye care.

Dimensions	Total (*n* = 248)	Pre-Visit Stage (*n* = 84)	Treatment Stage (*n* = 83)	Follow-Up Stage (*n* = 81)	*F*	*p*-Value
Attitude to health information	3.98 ± 0.75	3.81 ± 0.71	4.26 ± 0.65	3.88 ± 0.81	9.017	<0.001 ^a,c^
Information needs	4.15 ± 0.58	3.98 ± 0.43	4.36 ± 0.62	4.11 ± 0.61	10.637	<0.001 ^a,c^
Sources of information	3.43 ± 0.52	3.19 ± 0.46	3.53 ± 0.50	3.57 ± 0.53	15.024	<0.001 ^a,b^
Barriers to accessing health information	2.47 ± 0.73	2.69 ± 0.76	2.45 ± 0.62	2.27 ± 0.73	7.445	0.001 ^b^
Overall	3.56 ± 0.32	3.44 ± 0.25	3.70 ± 0.32	3.55 ± 0.34	17.443	<0.001 ^a,c^

^a^ Pre-visit stage versus treatment stage, Bonferroni-corrected *p* < 0.05; ^b^ Pre-visit stage versus follow-up stage, Bonferroni-corrected *p* < 0.05; ^c^ Treatment stage versus follow-up stage, Bonferroni-corrected *p* < 0.05.

**Table 3:** Binary logistic regression of health information-seeking behavior for different eye care stages.

Variables		Odds Ratio (95% CI)	*p*-Value
Pre-visit stage			
Monthly income (CNY, ¥)	≤2000	1 [Reference]	
	2001~6000	3.73 (1.08, 12.91)	0.037 *
	>6000	1.98 (0.57, 6.91)	0.283
Self-reported overall health	Good	1 [Reference]	-
	General	3.05 (0.80, 11.64)	0.102
	Poor	5.10 (1.46, 17.90)	0.011 *
Internet access	Yes	1 [Reference]	-
	No	0.17 (0.04, 0.68)	0.013 *
Treatment stage			
Education level	Primary school or below	1 [Reference]	-
	Middle school	1.23 (0.31, 4.86)	0.764
	High school or above	6.45 (1.34, 30.98)	0.020 *
Place of living	Urban	1 [Reference]	-
	Town	0.66 (0.15, 2.87)	0.578
	Rural	0.24 (0.04, 1.31)	0.098
Monthly income (CNY, ¥)	≤2000	1 [Reference]	-
	2001~6000	0.13 (0.03, 0.57)	0.007 *
	>6000	0.22 (0.03, 1.39)	0.107
Categories of VI	Unilateral	1 [Reference]	-
	Bilateral	0.16 (0.03, 0.81)	0.026 *
VI in the better eye	None	1 [Reference]	
	Mild	0.11 (0.02, 0.62)	0.013 *
	Moderate to blindness	0.16 (0.03, 0.81)	0.026 *
Self-reported overall health	Good	1 [Reference]	
	General	5.40 (0.97, 29.93)	0.054
	Poor	7.68 (1.45, 40.64)	0.017^*^
Follow-up stage			
Place of living	Urban	1 [Reference]	-
	Town	1.01 (0.24, 4.20)	0.989
	Rural	8.18 (1.15, 57.91)	0.035 *
Primary caregiver	Others	1 [Reference]	
	Spousal caregiver	6.36 (1.14, 35.40)	0.035 *
	Child caregiver	10.64 (2.14, 53.02)	0.004 *
Self-reported overall health	Good	1 [Reference]	-
	General	13.74 (1.89, 99.79)	0.010 *
	Poor	67.81 (9.73, 472.69)	<0.001 *

* represent the significant differences (*p* < 0.05), Abbreviations: Cl, confidence interval; VI, vision impairment.

## Data Availability

The data presented in this study are available on request from the corresponding author due to privacy and legal reasons.
